# Spun Biotextiles in Tissue Engineering and Biomolecules Delivery Systems

**DOI:** 10.3390/antibiotics9040174

**Published:** 2020-04-12

**Authors:** Catarina S. Miranda, Ana R. M. Ribeiro, Natália C. Homem, Helena P. Felgueiras

**Affiliations:** Centre for Textile Science and Technology (2C2T), Department of Textile Engineering, University of Minho, Campus of Azurém, 4800-058 Guimarães, Portugal; catarinanda@gmail.com (C.S.M.); rita.ribeiro_02@hotmail.com (A.R.M.R.); natalia.homem@outlook.com (N.C.H.)

**Keywords:** regenerative medicine, tissue engineering, micro- and nanofibers, soft and hard tissue substitution, local and systemic biomolecule delivery

## Abstract

Nowadays, tissue engineering is described as an interdisciplinary field that combines engineering principles and life sciences to generate implantable devices to repair, restore and/or improve functions of injured tissues. Such devices are designed to induce the interaction and integration of tissue and cells within the implantable matrices and are manufactured to meet the appropriate physical, mechanical and physiological local demands. Biodegradable constructs based on polymeric fibers are desirable for tissue engineering due to their large surface area, interconnectivity, open pore structure, and controlled mechanical strength. Additionally, biodegradable constructs are also very sought-out for biomolecule delivery systems with a target-directed action. In the present review, we explore the properties of some of the most common biodegradable polymers used in tissue engineering applications and biomolecule delivery systems and highlight their most important uses.

## 1. Introduction

For many years, the use of artificial fibrous structures was restricted to applications in clothing and decoration. In the past century though, fiber constructs made breakthroughs in engineering with applications in filtration, composite fabrication, energy systems and microfluids [1]. More recently, fibers manufactured as mono- or multi-filaments entered the field of medicine.

Biotextiles based on natural and synthetic fibers are very common in tissue engineering. They are defined as ‘‘structures composed of textile fibers and designed for uses in a specific biological environment (e.g., surgical implant), where their performance depends on their interactions with cells and biological fluids as measured in terms of biocompatibility and biostability’’ [2]. The applications of biotextiles cover a large spectrum; they have been used in heart valve swing rings, vascular grafts, hernia repair meshes, percutaneous access devices, wound dressings, delivery systems, etc. [3]. In many of these cases, biotextiles are processed in the form of fibrous structures with a large surface area and adequate chemistry, high and uniform porosity and pore interconnectivity, resembling the fibrous architecture of the extracellular matrix and, in this way, enabling cell adhesion and migration, vascularization and nutrients transport [4,5].

Manufacture of biocomposites can be achieved by several processing techniques, which allow biotextiles, presented in the form of loose fibers, nonwoven mats, aligned yarns or woven fabrics, to attain specific mechanical properties in the final product. Other factors influencing their properties are the type of natural or synthetic fiber, the chemical compatibility between the fiber and matrix phases, the corresponding surface energies and the properties of the interface. Furthermore, fiber separation and extraction processes play a determinant role in the quality, yield and structure of the final product [5].

Polymer fibers used in tissue engineering applications and biomolecule delivery systems can be fashioned using a variety of additive manufacturing techniques, such as 3D printing. However, the most common are the spinning methods: wet spinning, dry spinning, melt spinning, gel spinning and electrospinning (co-spinning and co-axial spinning being the most recurrent for delivery systems) [1,6]. All of these are based on the extrusion of polymer melts or solutions through a spinneret under controlled operating, solution and environment conditions. During fiber processing, using biodegradable polymers, additional monitoring is required to prevent polymers from undergoing degradation. This is particularly important during melt processing due to the high temperatures [7]. Constructs based on biotextiles should be capable of restoring the local biomechanical functions while maintaining a controlled degradation rate that matches the tissue healing or regeneration processes [3]. Thus, fabrication of fibrous devices that support and instigate tissue regeneration using biodegradable polymers endowed with biocompatibility and biostability properties is recommended for successful outcomes. For regeneration purposes, it is also necessary to select the appropriate cell sources. Mesenchymal stem cells are frequently selected due to their unique capacity of differentiation into one or several types of specialized cells [8]. In biomolecule delivery systems, the compatibility of the drug (antibiotic, growth factors, antimicrobial peptide, essential oil, plant extract, etc.) with the polymer is crucial for a successful outcome [5,9,10]. Still, challenges remain on this front due to the degradation or denaturation of the biological molecules when combined with organic solvents.

Previous publications focused on the advances in the manufacturing of biofunctional fibers [11] and on the development of nanofibrous scaffolds by electrospinning [12], natural-fiber reinforced biocomposites [5] and cellulose-based electrospun scaffolds for wound healing [13], as well as possible tridimensional fibrous structures for flat bone regeneration [14] and even the use of electrochemically aligned collagen threads in the form of scaffolds to repair critical infraspinatus tendon defects [15]. The present work addresses these issues and the applications and advantages introduced by biotextiles in tissue engineering and controlled drug release. In fact, here, the properties of the most commonly used natural and synthetic biodegradable polymers in tissue engineering and biomolecule delivery systems and their processing technologies are enumerated. Development and optimization of biodegradable fibers and the respective processing conditions are discussed in light of their final application, and important outcomes introduced by such systems are reviewed. Finally, examples of biodegradable biotextiles applied in soft and hard tissue repair and substitution and in biomolecule-controlled delivery applications are also highlighted.

## 2. Biodegradable Polymers

For many years now, biodegradable polymers (hydrolytically and enzymatically degraded) have been used for biomedical applications with an emphasis on tissue engineering and biomolecule delivery systems for regenerative medicine. It is predicted that in the next few years, they will gain an even more important status by replacing biostable temporary therapeutic devices used only for substitution. In light of the ethical and technical issues that surround the latter, which most of the time require revision surgeries, the use of biodegradable polymers, capable of stimulating the body to repair and regenerate damaged tissues, is desirable [16,17].

In regenerative medicine, biodegradable polymers are required to exhibit specific properties: (1) they do not instigate or induce a toxic response upon implantation; (2) they present an acceptable shelf-life; (3) they degrade at a rate that matches the healing or regeneration processes; (4) they possess appropriated mechanical properties that vary with degradation in a proportion compatible with the healing or regeneration processes; (5) they do not produce toxic degradation by-products; and (6) they are permeable and easily processed for the intended application [18]. It should also be noticed that the chemical, physical, mechanical and biological properties of biodegradable polymers may alter during degradation and thus should be monitored over time [16].

Degradation of synthetic and natural polymers requires cleavage of bonds sensitive to hydrolytic or enzymatic action. Synthetic polymers are characterized by predictable properties and a certain uniformity in site-to-site and patient-to-patient outcomes. They can be processed with specific properties that respond to local demands or application requirements and are most of the time hydrolytically degraded. Compared to polymers susceptible to enzymatic degradation, those hydrolytically degraded are preferred due to the predictability in body response after implantation [16]. Table 1 provides a list of some of the most common synthetic biodegradable polymers used in tissue engineering and their inherent properties, including average degradation rates (time to complete resorption).

### 2.1. Synthetic-Origin Polymers

Several synthetic polymers have been fabricated and explored over the years, mostly by polymerization techniques. The majority provide unique and important physical and chemical properties, and some of them are applied in the design of scaffolds for drug delivery systems and tissue engineering purposes. There are different kinds of synthetic biodegradable polymers, such as polylactic acid (PLA), polyglycolic acid (PGA), poly(ε-caprolactone) (PCL), which have demonstrated exceptional biocompatible features such as degrading in vivo into non-toxic components at controlled rates and to displaying good mechanical properties [22].

#### 2.1.1. PCL

PCL is a hydrophobic, semi-crystalline, linear resorbable aliphatic polyester obtained by either ring-opening polymerization of caprolactone or via free-radical ring-opening polymerization of 2-methylene-1-3-dioxepane [23]. Its biodegradation is associated with its aliphatic ester linkage susceptibility to hydrolysis. The good solubility, low melting point and compatibility with other polymers make PCL exceptionally great for biomedical applications [24].

#### 2.1.2. PGA

Poly(glycolic acid) (PGA) is a highly crystalline, biocompatible polyester with good mechanical features and degradation profile, low solubility in organic solvents and excellent fiber-forming ability due to its high tensile modulus [25,26]. PGA was used in the production of the first synthetic, absorbable surgical suture [27]. However, because of its hydrophilic nature and quick water uptake, its mechanical strength may be lost after a period of 2 to 4 weeks post-implantation. Since then, PGA has been used for a variety of purposes, including bone fixation devices, biomolecule carriers and scaffolds matrices for tissue regeneration [19,25,26].

#### 2.1.3. PLA

PLA is a biodegradable, aliphatic polyester derived from lactic acid. It is a versatile polymer made of renewable materials, i.e., corn starch or sugar cane, that can be fermented into lactic acid and prepared via cyclic dilactone, lactide, ring-opening polymerization. During this process, PLA enhances its thermal stability, stiffness and strength and reduces residual monomer content [28]. PLA is more hydrophobic than PGA due to the presence of an extra methyl group in lactic acid [29].

#### 2.1.4. PLGA

By varying the ratios between its copolymers PGA and PLA, the poly(lactic-co-glycolic acid) (PLGA) co-polymer offers a wide range of degradation rates. Its degradation kinetics is governed by both the hydrophobic/hydrophilic balance and crystallinity, which makes PLGA particularly desirable for tissue engineering applications due to its excellent biocompatibility and biodegradable properties [20,30].

#### 2.1.5. PDLA, PLLA and PDLLA

**Figure 1:**
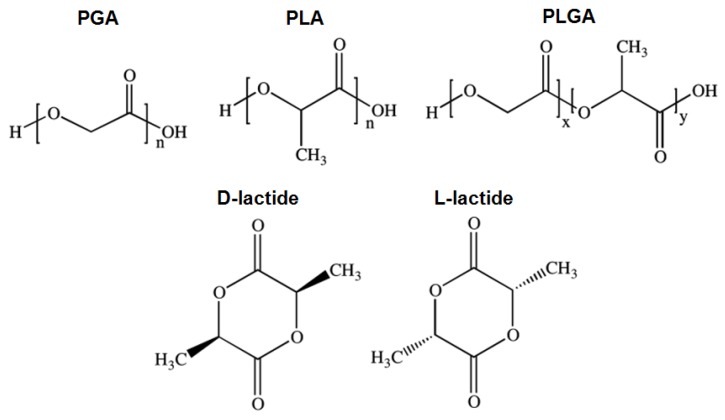
Chemical structure of PGA, PLA, PLGA and the enantiomers D- and L-lactide.

PLA exists in two stereoisomeric forms, giving rise to PDLA and PLLA, two stereoregular polymers, and PDLLA, a racemic polymer obtained from the mixture of D- and L-lactic acid (Figure 1). PDLLA is an amorphous polymer commonly used for drug delivery due to its ability to disperse homogeneously the active species within a monophasic matrix. As semi-crystalline polymers, both PDLA and PLLA are favored for applications in orthopedics where high mechanical strength and toughness are necessary [21,31,32].

#### 2.1.6. PDO

Unlike the above, the biodegradable aliphatic polyester polydioxanone (PDO) has received limited interest until recently. It was first used in 1981 as a monofilament suture [33] but only now has been applied to other uses, including stents, rings for pediatric mitral and tricuspid heart valve repair and as plates for orbital floor reconstruction [34]. PDO presents a good safety profile with low toxicity in vivo and is capable of completely degrading between 6 and 12 months, depending on its degree of crystallinity, which also affects the polymer absorption rate. Compared to PGA, PLA and their derivatives, PDO displays a lower tensile modulus with limited mechanical performance. However, is still suitable for a wide range of tissue engineering applications like cartilage, ligament and vascular tissue engineering [35,36].

### 2.2. Natural-Origin Polymers

Regarding natural polymers, the majority degrade very quickly via enzymatic degradation. The rate of degradation depends on the implantation site, accessibility to and concentration of enzymes and possible chemical alterations made to their structure. They are also susceptible to cell-triggered proteolytic degradation [16]. Natural polymers have been used for many years in tissue engineering. They are classified as polysaccharides, polymeric carbohydrate molecules formed of glycosidic-bound monosaccharide units such as alginate, hyaluronic acid, cellulose and chitosan, and as polypeptides, peptide bound amino acid chains that include collagen and gelatin, for instance [37,38,39].

#### 2.2.1. Alginate

Alginate is a polyanion, typically obtained from brown seaweed, that possesses solubility in water, great biocompatibility and low toxicity and is biodegradable and of relatively low cost. Most alginates can be processed in the form of hydrogels, porous scaffolds, microparticles and nanoparticles [40]. Alginates are readily degraded by naturally occurring enzymes, i.e., lysases. Their physical and mechanical properties are dependent on the length, molecular weight and proportion of the guluronate block within the polymeric chain [41,42]. Alginates can be prepared by various cross-linking methods in a similar way to the extracellular matrix of living tissue, making them desirable for applications in wound healing, drug delivery, and cell transplantation [40,43].

#### 2.2.2. Hyaluronic Acid

Hyaluronic acid is a glycosaminoglycan made up of repeating disaccharide units of D-glucuronic acid and β-1,3-N-acetyl-D-glucosamine. It is commonly found in conjunctive tissues of any vertebrate and can be rapidly biodegraded by the human body [44]. Hyaluronic acid is also a polyanion that can self-associate and bind to water molecules, giving it a stiff, viscous quality similar to gelatin [45]. Because of its unique rheological properties and complete biocompatibility, hyaluronic acid has been used quite extensively in biomedical applications, playing a significant role as an antifouling agent protecting blood-contacting devices, in wound healing, biomolecule delivery and tissue regeneration [46,47].

#### 2.2.3. Cellulose

Cellulose is an abundant polysaccharide based on glucose and is present in plants, bacteria, fungi, algae and animals. Cellulose can also be biosynthesized by bacteria in the form of bacterial cellulose. It exhibits a unique nanostructure, remarkable physical-chemical properties and biocompatibility and is resistant to hydrolysis, strong alkali and oxidizing agents [48,49]. Cellulose on its own cannot be electrospun. However, each repeated unit of cellulose possesses three hydroxyl groups on its structure, which can be replaced by other chemical groups, such as methyl, acetyl, and carboxymethyl, and thus, several derivative compounds can be synthesized [50]. The acetate ester of cellulose, also known as cellulose acetate, is the most commonly used. Due to its unique properties such as high tensile and impact strength, good antistatic properties, good transparency, excellent scratch resistance, high moisture absorption, and permeability, cellulose esters have found numerous applications in biomedicine [13,26,51,52].

#### 2.2.4. Chitosan

Chitosan is the most widely used natural polymer in drug delivery due to its ability to blend with a variety of synthetic polymers and facile surface modification. Derived from partially deacetylated chitin found in the shell of crustacean, cuticles of insects and cell walls of fungi microorganisms, it is composed of D-glucosamine and N-acetylglucosamine repeat units, forming the only pseudonatural cationic polymer. By varying the degree of deacetylation, viscosity and molecular weight, a series of chitosan polymers may be generated [53,54]. The degree of deacetylation of typical commercial chitosan is usually between 70% and 95%, and the molecular weight can range from 10 to 1000 kDa. These three factors (degree of deacetylation, viscosity and molecular weight) are also determinant to the polymers’ physical and chemical properties. Regardless, all chitosan polymers are known for their biocompatibility, biodegradability, antimicrobial activity, wound healing abilities and antitumor effect [55]. Chitosan solubility in aqueous solutions makes it processing into gels, films or fibers possible [56,57,58].

#### 2.2.5. Collagen

Collagen is the most abundant mammalian protein, accounting for around 30% of all body proteins, and is a major component of ligaments, tendons, skin and bone [59,60]. In its native environment, collagen interacts with cells in connective tissues and transduces essential signals for the regulation of cell anchorage, migration, proliferation, differentiation and survival. It is composed of polypeptide strands bearing triamino acid blocks of glycine-X-Y, where X and Y can be any of a number of amino acids, that organize themselves into left-handed triple helix microfibrils [61]. Collagen is a good surface-active agent and is capable of penetrating within lipid-free interfaces. It is desirable in many biomedical applications because of its biodegradability, high mechanical strength, weak antigenicity and superior biocompatibility compared with other natural polymers, i.e., gelatin [60,62,63,64]. Electrospinning of soluble collagen is a suitable method to prepare scaffolds with high porosity and surface area for tissue engineering [58].

#### 2.2.6. Gelatin

Gelatin is a natural polymer derived from the controlled structural and chemical degradation of collagen and contains a large number of glycine, proline and 4-hydroxyproline residues. Gelatin comprises many functional groups and cell-binding sites in its structure, increasing its cell-binding ability and making it desirable for tissue engineering applications. It is also commonly used to produce biocompatible and biodegradable biomolecule delivery systems and wound dressings. In fact, targeted tissues include bone, cartilage and skin, but others such as adipose tissue have also applied gelatin as a carrier for the delivery of active biomolecules using the process of encapsulation to improve temporary cell functions [58,65,66].

### 2.3. Bio-Synthetic Hybrid Polymers

As seen, the most relevant properties of natural polymers are their bioactivity (biological recognition), biocompatibility, antigenicity and non-toxicity (which reduces undesirable host responses), tunable degradation kinetics and, in many cases, presence of cell-recognition sites. However, these materials may also display limitations that include weak mechanical strength, rapid or unregulated degradation rate and inconsistency in composition (lack of reproducibility of properties between batches) due to their natural source. In turn, synthetic polymers are easily processed at a large scale, are relatively low cost and display controlled properties, including molecular weight and functionality. Despite these important advantages, one main drawback haunts synthetic polymers and restricts their application in biomedicine, namely the inability to be recognized by cells and consequently induce their adhesion and proliferation [67].

The rationale behind preparing hybrid polymers instead of using single-component polymers is to combine the positive functionalities of both natural and synthetic to generate a construct that exceeds the individual properties of each individual polymer composing the hybrid [68]. Synthetic polymers with good mechanical properties but lacking motifs for cell recognition, attachment or proliferation can be combined with small amounts of natural-origin polymers to enhance these properties and, this way, generate a material with easier processability. Even though these modifications may lead to better-performing materials for applications in tissue engineering or drug delivery systems, synthesizing hybrid materials can be very challenging, especially when different fabrication techniques must be employed. Therefore, a balance between the complexity of the production and fabrication processes and the benefits presented by the hybrid materials must be maintained [69,70].

Tissue-engineered scaffolds are being developed as treatment options for malfunctioning tissues throughout the body. Therefore, it is essential for the scaffold to closely mimic the native tissue with regards to both mechanical and biological functionalities. For instance, the use of synthetic polymers modified with collagen increases the scaffold mechanical strength. Moreover, loading bioactive molecules, such as growth factors, into collagen-based scaffolds and gels enhances their regeneration and restorative effects. The main challenge in drug delivery systems made of hybrid polymers remains the liberation of selected biomolecules at specific targets. The dominant route for drug administration is through oral routes or intravenous injections, but these methods have limited access over the drug release rate in the body as they show a tendency for immediate burst release. To overcome this issue, hybrid polymers engineered with chemically modified chitosan or cellulose are finding applications in multiple areas, including cell encapsulation, wound dressings and implants, with effective outcomes [13,58,71,72].

## 3. Biotextiles Production: Fiber Technologies

The history of fiber production by humankind can be traced back to prehistoric times. Fragments of cotton articles dating back to 5000 BC have been excavated, and silkworm cultivation began in 2700 BC to produce silk fibers and textiles. Around 1300, the spindle was invented to fabricate fibers from wool and cotton used in fabrics and clothes, and this practice slowly evolved into the textile industry in the 1880s. About 50 years later, production of synthetic fibers initiated and with that the development of chemistry and polymer sciences [73].

Nowadays, several fabrication methods can be applied to convert polymers into fibers. The resulting fibers can either be continuous monofilament yarns or multifilament yarns or, alternatively, they can be divided into short-length staple fibers and blended with natural fibers such as cotton or wool or used by itself in the manufacture of scaffolds. To this aim, 3D printing and fiber spinning technologies can be regarded as the most prominent techniques in this field [74,75].

### 3.1. Fiber Extrusion Spinning

Great progress in spinning polymer fibers for biotextiles has been achieved over the past few decades [76]. Spinning is a specialized form of extrusion that uses a spinneret to form mono or multiple continuous filaments and is considered an interdisciplinary technique that applies the principles of engineering and material science. The three principal spinning methods conventionally used to manufacture fibers are wet-spinning, dry-spinning and melt-spinning [77,78].

The first step to producing fibers by spinning techniques is to convert the polymer into a processable and spinnable state. Thermoplastic polymers can be converted into a fluid state and melt-spun [79]. Other polymers may be dissolved in a solvent or chemically treated to form soluble or thermoplastic derivatives and subsequently spun via dry-spinning, wet-spinning or electrospinning, a most recent approach to spinning at the nanoscale. The main traditional spinning approaches used in the fabrication of biotextiles are introduced in the following sections.

#### 3.1.1. Melt-Spinning

Melt-spinning is the most economical spinning process as it does not require solvents to process the polymers. For that reason, it is the preferred method for manufacturing polymeric fibers, being extensively used in the textile industry. However, this technique presents limitations to its use in the production of biostructures, including decomposition at temperatures below the melting point, poor control over the exact temperature of the polymer melt during spinning, thermo-mechanical history of the melt and final fiber structure/morphology [80].

In the melt-spinning process, dried polymer granules or chips are melted inside the extruder in order to obtain the spinning dope. This viscous melt is then extruded through a spinneret, and the obtained filament is quenched and solidified by cooling in a fast fiber solidifying process [81]. Despite its limitations, melt-spinning of biopolymers from different sources has been extensively explored in bio-applications (Table 2) [82]. The use of bio-based reinforcements is reported as an alternative to solve the problems associated with the synthesis of biotextiles via melt-spinning [83].

#### 3.1.2. Dry-Spinning

Dry-spinning is one of the oldest spinning methods in use. Contrary to melt-spinning, in dry spinning, the polymer is dissolved in an appropriate solvent. The polymer solution is then extruded through a spinneret, subsequently passing through a heating column, where the solvent evaporates, leaving behind dry fibers (in this case, highly volatile solvents are required). In the heating column, steam of hot air or inert gas is used to solidify the fibers and remove the solvent. This technique is suitable for polymers vulnerable to thermal degradation and cannot form viscous melts, and for when specific surface characteristics are required from fibers. Several polymer fibers have been traditionally processed using dry-spinning techniques, including acetate and triacetate, some modifications of acrylics and modacrylics, aramid fibers and spandex fibers [77,78]. Apart from the recovery process, the mass transfer mechanisms involved in the solvent evaporation and filament formation make dry-spinning more complex and expensive than melt-spinning [79].

#### 3.1.3. Wet-Spinning

The first fiber to be spun by wet-spinning was rayon, with an alcoholic solution of cellulose nitrate extruded from a nozzle dipped in cold water. Since then, many natural and synthetic polymers have been produced via wet-spinning [94]. Like dry-spinning, in the wet-spinning technique, the polymer needs to be dissolved in a suitable solvent in order to be spun. However, here, the solvent does not need to be volatile [95].

Wet-spinning is based on the principle of precipitation, in which a phase inversion occurs during the extrusion of the polymeric solution through a spinneret directly into a coagulation bath composed of a non-solvent liquid [75]. Natural-origin polymers such as gelatin, alginate, collagen, cellulose and many of their composites have been processed in the form of fibers using wet-spinning for a variety of tissue engineering uses (Table 2). This technique allows the production of fibers with relatively large diameters (in the nano-to-micrometer ranges) and the construction of architectures with high porosity and interconnected open pore structure, which are desirable for cell penetration, adhesion and proliferation [96].

#### 3.1.4. Electrospinning

Electrospinning is a unique spinning approach that resorts to electrostatic forces to produce fine fibers (from nanometer to micrometer) from polymer solutions or melts with a larger surface area than those obtained from conventional spinning processes [97].

Electrospinning has attracted much attention in biomedical research because of the inherent properties of the resultant nanofibers, such as large surface areas, high porosity and a continuous three-dimensional web structure [6,39,98]. The process involves the ejection of a polymer solution through a needle, termed the spinneret, that under an electric field is attracted towards a collector plate. A high potential difference is applied between the two, resulting in the conversion of the initial solution into nanofibers [39,99,100].

Nanofibrous scaffolds have shown great potential and usefulness for assisting the regeneration and reconstruction of different types of human tissues and organs, ranging from bone, skin and blood vessels to organs like liver and kidneys [101]. Early research revealed that electrospun nanofibrous scaffolds of biodegradable polymers could closely mimic the hierarchical architecture of native extracellular matrix and facilitate good attachment and proliferation of cells. Later, the potential of highly biocompatible electrospun scaffolds for engineering many types of human body tissues with relatively simple structures was shown and is now frequently realized [102].

### 3.2. 3D-Printing

Printing methods have raised great attention in recent years as fast and inexpensive ways of manufacturing several materials. The 3D printing technique, which is an additive manufacturing technique, was initially conceived in the 1980s. Since then, this technique has impacted many fields, entering just recently the biomedical area [103]. 3D printing of complex biomedical devices designed using patient data is an expanding field of research, and applications can range from the reconstruction of complex organs with intricate 3D microarchitecture (e.g., liver, lymphoid organs) to scaffolds for stem cell differentiation [104].

In general, 3D printing includes different printing technologies to generate 3D structures by adding layers upon layers of materials, such as ceramics, metals or polymers (synthetic or natural) [105]. The standard for 3D printing technologies (ASTM F2792) is applied to several rapid prototyping processes such as vat photopolymerization, material jetting, material extrusion, powder bed fusion, binder jetting, sheet lamination and directed energy deposition. The vat photopolymerization 3D printing method has a container with a photopolymer resin hardened by means of a power source (e.g., ultraviolet light). Here, the most common technique employed is the stereolithography (SL) [106,107]. In bioprinting, SL can generate multiple cycles to form a 3D structure by photopolymerization/cross-linking of polymers [103]. The main disadvantage of using such technology for biomedical purposes is the need for intense ultraviolet radiation during cross-linking. Other limitations are the lengthy post-processing time requirement and the lack of diversity of biomaterials that can be applied as “biomaterial inks” (bioinks) [108]. Gelatin- and alginate-derived bioinks, for instance, suffer from poor shape fidelity and poor printing resolution and form very soft gels at physiologic temperatures [109]. Strategies to improve these limitations have been employed, such as introducing new functional groups via crosslinking and forming composites of natural and synthetic bioinks. These efforts have been rewarded with an improved printing fidelity, resolution and mechanical integrity that does not interfere with the inherent biocompatibility of natural bioinks [110].

Another potential application of 3D bioprinting is in tissue replacement and restoration using seeded stem cells. One of the major advantages of bioprinting compared to conventional tissue engineering strategies is the ability to influence stem cell differentiation at multiple stages. The choice of stem cell source, bioprinting method, scaffold architecture, additives used and mechanical forces applied can influence stem cell differentiation towards a specific target tissue [103].

## 4. Tissue Engineering

In tissue engineering, principles from biology, chemistry and engineering sciences are combined towards the common goal of regeneration. Engineering living systems for regeneration purposes requires appropriate cell sources, optimal culture conditions, and biodegradable implantable devices. In the first two cases, consensus has been reached by recommending the use of specialized cells or mesenchymal stem cells capable of differentiation, and bioreactors to provide optimal and controlled culture conditions. Regarding the biodegradable implants, the construct is expected to guide cell growth and tissue formation with time in three dimensions. As described in Section 2, there are many polymers capable of such a task [6,39,111]. Fibers and biotextiles have been successfully used in close contact with complex biological environments for a variety of applications, both by itself or loaded with specific biomolecules, due to their similarity with the tubular and fibrous architecture of many tissues, including muscle, tendon, ligament, bone and teeth [112]. In the following sections, examples of soft and hard tissue applications of fiber-based constructs in tissue engineering are provided.

### 4.1. Stents

Stents are small, expandable reticulated tubes with two main functions, the treatment of dissection and prevention of restenosis. Biodegradable stents are expected to provide a temporary opening of a narrowed arterial vessel until remodeling occurs and to disappear progressively thereafter. Indeed, an ideal biodegradable stent should be able to compromise its degradation rate and mechanical integrity during implantation and after it, during the 6 to 12 months expected for the remodeling process to be completed [113].

The first biodegradable stent was developed in 1988 by Stack et al. using PLLA and was almost completely degraded after 9 months. Clinical research on animal models revealed minimal presence of thrombosis, moderate neointimal growth and limited inflammatory response [114]. Later, it was demonstrated that low-molecular-mass PLLA was associated with intense inflammation, while the inverse was also true [115]. This was tested on different animal models: in dogs, minimal tissue growth was observed [116], while in pigs, marked cell proliferation occurred [117]. Yamawaki et al. were the first to incorporate an anti-proliferative agent on high-molecular-weight PLLA stents and to verify that neointimal formation was significantly reduced in the presence of the loaded tube. Nguyen et al. evaluated the hemocompatibility profile of PLLA stent fibers loaded with anti-inflammatory and anti-proliferative drugs, curcumin and paclitaxel by employing a closed-loop circulation system filled with human blood. Data revealed curcumin as more effective than paclitaxel, the activity of which may have been inhibited during melt extrusion, in reducing leukocyte and platelet adhesion and activation to the stent fibers [118]. More recently, Quin et al. used PDO monofilaments coated with small intestine submucosa to braid mesh stents. After 24 weeks implantation in dog animal models, the stents were completely degraded [119]. It has been shown that PDO filaments can lose up to 60% of their initial strength after 6 weeks of degradation in phosphate buffer saline solution, while preserving the stent well radial strength [120]. This was corroborated by Wang et al., whose PDO monofilaments braided in the form of a stent were capable of retaining half of their original strength after 6 weeks of degradation but lost it completely after 10 weeks. In the end, the stents demonstrated good compression performance up to 12 weeks and therefore could become a good alternative for short-term applications [121].

Genistein-conjugated PLLA has been synthesized by direct coupling with a unique influence on coagulation, plasma protein adsorption and subsequent platelet adhesion and activation. Genistein is a potential flavonoid endowed with anti-thrombotic and anti-proliferative properties, capable of inducing human platelet aggregation in a dose-dependent manner via nitric acid-dependent signal-transducing pathways. This fibrous system was designed for potential applications in coronary stents [122]. Drug loaded PLLA has also been conjugated with the two ends of the matrix metalloproteinase-9 (MMP-9) for a selective drug release, beneficial for stent endothelialization, thereby decreasing the risk of restenosis and thrombosis [123]. Tyrosine kinase inhibitor (ST638) was encapsulated onto prototype Igaki–Tamai stents and implanted in porcine coronary arteries. Data revealed the progressive, gradual release of the ST638 decreasing neointimal hyperplasia [117]. More recently, using a conventional emulsion solvent evaporation method, sirolimus-encapsulated PDLLA nanoparticles were produced and coated onto stents for a sustained biomolecule release. Cell culture studies demonstrated the ability of the fashioned stents to inhibit smooth muscle cell proliferation while accelerating endothelial cells, therefore unveiling the potential of these stents to decrease both the occurrence of in-stent restenosis and acute thrombosis [124].

### 4.2. Skin

**Figure 2:**
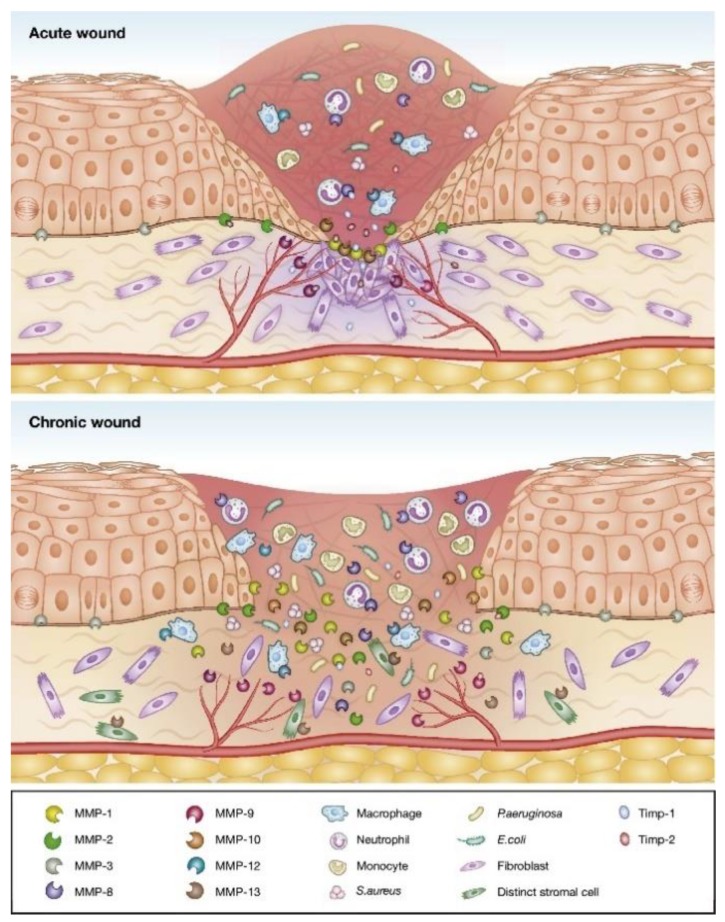
Depiction of acute and chronic wound scenarios with dysregulated matrix metalloproteinases (MMPs) and infiltration of bacteria (used with permission from [126]).

Skin is the largest organ in the body, formed mainly of two layers, the epidermis and the dermis, and primarily serves as a protective barrier against the environment. Skin wounds normally heal in a predictable amount of time by forming an epithelialized scar tissue. However, whenever burns, trauma, irreversible damaged skin or chronic wounds occur, the need for substitutes or more efficient protective barriers is raised (Figure 2) [39]. Engineered skin tissue and high-performance wound dressings would be an excellent solution. Although allografts and autografts have been the most obvious choice in the past, lately, temporary three-dimensional tissue engineering constructs mimicking the skin architecture and loaded with fibroblasts, keratinocytes and endothelial cells have gained more attention [10,125].

Biodegradable fiber-based structures have been proposed for the healing of dermal and epidermal injuries. In fact, PLGA fibrous dressings have been already successfully used in clinical patients diagnosed with diabetic foot ulcers (i.e., Dermagraft and Dermagraft-TC) [127]. Thin biodegradable hybrid meshes of PLGA and collagen have also been produced for culture of human skin fibroblasts. Results indicated the web-like collagen formations distributed along the PLGA knitted fibers increased the fibroblasts seeding and distribution and facilitated rapid formation of dermal tissue with uniform thickness [128]. Recently, Norouzi et al. produced nanofibers of PLGA and gelatin via electrospinning and demonstrated desirable bioactivity and hemostasis of the fibrous scaffolds with controlled release of the protein [129]. Electrospun PLGA nanofibers have also been encapsulated with epidermal growth factors (EGF), resulting in desirable biocompatible and bioactive scaffolds with EGF controlled release [130].

Collagen-based scaffolds have been the most popular for skin regeneration, both alone and in combination with other polymeric matrices or molecules (growth factors, proteins, etc.). For instance, dermal substitutes composed of type I collagen and elastin hydrolysate, applied in combination with split-skin mesh grafts, contributed to the healing of full-thickness wounds [131] and improved dermal regeneration [132]. It has also been shown that preparations of collagen type I and PCL result in optimal degradation kinetics and mechanical performance while supporting dermal fibroblasts attachment and proliferation. Developing a porous structure expedited the healing process, assisted in re-epithelialization and follicle regeneration, and promoted the formation of dermal tissue with a matrix architecture resembling normal, unwounded skin [133]. Additionally, they revealed that these microporous electrospun constructs pre-seeded with fibroblasts promoted greater wound healing than acellular scaffolds [134].

Nada et al. combined cellulose acetate with capsaicin, a pain-relieving drug, and sodium diclofenac, a nonsteroidal anti-inflammatory drug that reduces the action of substances responsible for causing pain and discomfort, in an electrospun mat and generated a controlled released system that accelerated patient relief [135]. To confer biocidal properties to cellulose acetate-based nanofibers, Jiang et al. modified their surface with 4,4’-diphenylmethane diisocyanate (MDI), with a complete death of bacteria colonizing the wound after a 30 min contact [136]. The improved synergistic effect of the oregano essential oil with cellulose acetate-based nanofibers against *Staphylococcus aureus*, *Escherichia coli* and the yeast *Candida albicans* infecting wounds has also been demonstrated and explained on the basis of the potent antimicrobial character of oregano oil’s molecular components carvacrol and thymol [137]. Cinnamon, lemongrass and peppermint essential oils loaded onto cellulose acetate electrospun mats have also shown similar outcomes. However, even though fibroblasts and human keratinocytes could attach and spread on the fibers’ surface, cell viability seemed to decrease with exposure time [138]. The anti-proliferative effect of essential oils against eukaryotic cells remains a challenge in tissue-engineering applications and biomolecule delivery systems.

As seen, synthetic biodegradable polymers such as PLA, PGA, PLGA and PCL have all been used as matrices for skin regeneration, both individually and in combination with other natural-origin polymers. They have been processed in the form of nanofiber matrices with large surface area, high wound exudates absorbency capacity, and high oxygen permeability. In addition, the small pore size has been regarded as an extra barrier to prevent bacterial penetration [39].

### 4.3. Nervous System

The nervous system is a complex organization of neurons or glial cells that regulate and coordinate body activities. Degeneration of these cells results in changes in the extracellular matrix of the neural tissue that trigger a variety of clinical disorders. Since neurons cannot regenerate by themselves, restoration of their functions by means of biological and synthetic tools, processed in the form of fibrous scaffolds or biomolecule delivery systems, is a priority [139,140].

Cells live in a complex mixture of pores, ridges and fibers in the extracellular matrix. Mimicking those conditions via porous, interconnected nanostructured constructs with large surface areas is recommended for the cells’ successful in-growth [39,141]. In fact, nerve stem cell differentiation and neurite outgrowth have been successfully supported by PLLA nanostructured porous scaffolds, demonstrating their potential cell carrier in nerve tissue engineering. Here, scaffolds are produced using advanced techniques in the form of complex guidance channels, which precisely mimic the natural repairing process in the human body [142]. The efficacy of PLLA nano- and microfibrous scaffolds produced via electrospinning was also examined under optimal conditions of fiber alignment and tailored diameters by culturing neural stem cells. Results showed the neural stem cells elongation and neurite outgrowth to be parallel to the direction of the PLLA fibers. The rate of differentiation was also higher on nanofibers than on microfibers [143]. Prabhakaran et al. developed PCL nanofibrous constructs and modified their surface by a simple plasma treatment. The goal was to improve Schwann cell adhesion, proliferation and interactions along the nanofibers for nerve tissue formation. After 8 days of culture, cells attached and proliferated on the surface-modified scaffold, expressing bipolar elongations and retaining their normal morphology. In addition, data showed this treatment to be more cost-effective than the production of PCL/collagen scaffolds, adding evidence to their potential to serve in peripheral nerve regeneration [144]. Reports on PCL/gelatin nanofibrous scaffolds have also shown to enhance nerve differentiation and proliferation and to support neurite outgrowth. Once again, the nerve cell elongation followed the alignment of the nanofibers and established this biocomposite suitability for nerve regeneration [145]. More recently, both PCL and gelatin were used for neural differentiation of human-induced pluripotent stem cells in the form of bi-electrospun nanofibers. The nanofibers supported the stem cells’ differentiation into neural cells and were able to improve the entire process [146]. The selection of PLLA, PLGA and PCL as base polymers to produce fibrous scaffolds for nerve tissue engineering is based on their mechanical stability, exceptional biocompatibility and, most importantly, their slow biodegradability. This allows for cells to adhere and differentiate into neural cells with no danger of losing their supporting matrix before the entire local neural network is restored.

Core-shell PLGA nanofibrous nerve guidance conduits loaded with nerve growth factors were fabricated via co-axial electrospinning and used to construct nerve guidance conduits for a 13 mm rat sciatic nerve defect. After 12 weeks of implantation, the aligned core-shell nanofibers promoted a superior functional recovery, with more nerve fibers being regenerated and displaying a more mature morphology than in the control group (bare PLGA) [147]. Co-axial nanofibers with poly(L-lactide-co-ɛ-caprolactone) (PLLACL, derived from the merge of PLLA and PCL) at the shell and bovine serum albumin combined with nerve growth factors at the core were produced with a sustained release that promoted the differentiation of rat pheochromocytoma cells (PC12). In co-axial systems, the biomolecules’ release profile can be finely tailored by modulating the morphology, porosity and composition of the nanofibers. Besides, diameters at nanoscale provide short diffusion passage length and a high surface area very effective for controlled mass transfer [148]. Nerve growth factors have also been loaded onto PLLACL/silk fibroin solutions to produce co-axial fibers with a sustained release that lasted over 60 days. Here too, nerve guidance conduits were fabricated by reeling the aligned PLLACL/silk fibroin nanofibers and implanting these structures onto sciatic nerve defects in rats for 12 weeks. In the end, data demonstrate the ability of these structures to promote peripheral nerve regeneration by the local, controlled release of nerve growth factors [149]. PLLACL/silk solutions have been modified with Vitamin B5 to produce aligned nanofiber meshes; 80% of the Vitamin B5 content was released within 24 h, demonstrating this drug delivery system strategy’s potential to increase cell survival and proliferation and, ultimately, for applications in nerve repair or regeneration [150]. In another strategy, PCL and chitosan were blended to form nanofibrous scaffolds, via electrospinning, with excellent mechanical and surface properties. The surface of the scaffolds was functionalized with laminin via carbodiimide-based cross-linking. Data reported the successful growth and proliferation of Schwann cells within the laminin-loaded polymeric matrix and its versatility for in vivo cell delivery for nerve tissue engineering [151]. The important role played by PCL and PLLACL in nervous system research has been extensively confirmed. In fact, its use as a suitable platform for neuronal cell growth and proliferation is here extensively demonstrated. However, on its own, these polymers are barely effective, requiring growth factors and other biomolecules to be loaded onto their structure to stimulate the development and growth of the neural cells.

### 4.4. Vascular Grafts

**Figure 3:**
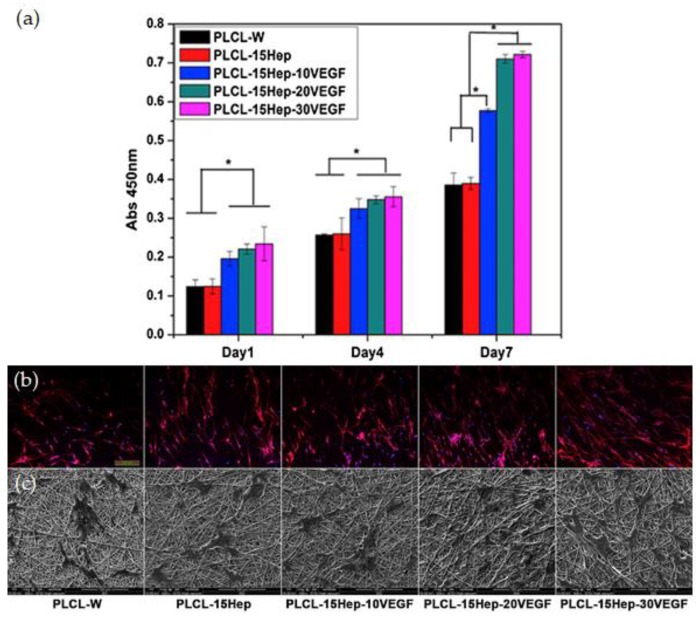
(**a**) Endothelial progenitor cell growth from 1 to 7 days (PLCL-W, control without biomolecules; 15Hep, 15 wt% heparin; 10, 20 and 30 vascular endothelial growth factors (VEGF) represent 10, 20 and 30 μg/mL of VEGF) and visual detection of the cells via (**b**) immunofluorescent microscopy (scale 200 µm) and (**c**) scanning electron microscopy (SEM, scale 100 µm) (adapted with permission from [162]).

Cardiovascular diseases are the number one cause of death globally. They are commonly associated with the narrowing blockage of blood vessels leading to reduced blood flow and inadequate nutrient supply. Synthetic vascular grafts have become a recurrent alternative to replace or bypass a damaged or occluded vessel [152]. In the first attempts to develop vascular grafts, non-biodegradable polymeric fiber structures made of Dacron and Teflon were employed [153]. Only later, arterial regeneration was demonstrated over woven PGA biodegradable constructs in rabbit models [154] or on microporous, compliant, non-thrombogenic L-polylactide-polyurethane (PLLA-PU) [155]. From then on, many different studies were conducted to improve the properties of biodegradable scaffolds and develop blood vessel substitutes. The goal was to engineer biodegradable grafts to serve only as skeleton constructs to induce and support tissue overgrowing and ingrowing. For instance, Sell et al. designed a PDO and elastin vascular graft via electrospinning, conducive to tissue regeneration, with mechanical properties that closely matched the native arterial tissue. They determined that a 50/50 ratio between the two biodegradable polymers was preferred to mimic the compliance of the native femoral artery and established elastin-containing grafts as bioactive, promoting cell migration [156]. Jeong et al. created a novel tubular scaffold from marine collagen and PLGA fibers that improved the mechanical strength of the collagen-based synthetic grafts in both dry and wet states. The proliferation and phenotype expression of smooth muscle cells and endothelial cells was followed on static and dynamic conditions. It was observed that under a pulsatile perfusion system, both cell proliferation and alignment along the PLGA fibers was improved and that cells were capable of retaining their differentiated cell phenotype [157]. A bi-layer of type I collagen was also tested in vivo as a small diameter vascular graft to promote the complete regeneration of rats’ inferior vena cava. After 12 weeks of implantation, a thin continuous layer of endothelial cells and smooth muscle cells was lined with the vascular lumen and tunic media, proving that the engineered vascular substitute not only possessed sufficient tensile strength and good biocompatibility but also advanced vascular regeneration [158]. More recently, PCL micro- and nanofibrous vascular grafts were engineered via electrospinning, and their long-term performance was investigated in vivo. PCL grafts were implanted in rat models at the abdominal aorta region up to 18 months, and their compliance, tissue regeneration and degradation rate were followed. Results showed excellent structural integrity, with no aneurysmal dilation, and perfect patency with no thrombosis and limited intimal hyperplasia. Cell migration and neovascularization increased quickly throughout time. Degradation data was inclusive [159]. In another in vivo study, PCL scaffolds were prepared with thicker fibers and larger pore structure and implanted in the abdominal aorta of rat models for 100 days. The macroporous vascular grafts enhanced cell infiltration and extracellular matrix secretion. At the end of the implantation period, endothelium coverage was complete, and the regenerated smooth muscle layer was correctly organized. More importantly, the regenerated arteries demonstrated a contractile response to adrenaline and acetylcholine-induced relaxation. These thicker-fiber electrospun scaffolds were also seen to attract and mediate macrophage polarization into the immunomodulatory and tissue remodeling phenotype [160]. To balance the degradation rate of PCL, fibrous grafts have also been prepared from blends of PCL and PDO. Pan et al. produced small-diameter hybrid grafts by co-spinning and followed their regeneration abilities in rat abdominal aorta replacement models up to 3 months of implantation. Degradation of PDO provided extra space within the graft, which facilitated vascular smooth muscle regeneration within PCL/PDO grafts. Coverage by endothelial cells was superior on PCL/PDO than on PCL grafts due to the increase in the construct’s hydrophilic nature. Heparin-loaded PCL nanofibers have also been prepared via co-axial electrospinning. An initial burst release of heparin (50%) was observed, followed by a more gradual release of up to 72% of its content within the following 2 to 14 days. Evaluation with a canine artery model revealed the potentialities of this system to greatly enhance the patency rate of small-diameter grafts [161]. Heparin has been combined with vascular endothelial growth factors (VEGFs) and loaded onto PLLACL (or PLCL), electrospun nanofibers for anticoagulation and rapid endothelization purposes. After adding Span-8 to the mixture, the synergistic action of heparin and the VEGFs was enhanced with a sustained release for 29 days that improved the scaffold anticoagulation capacity and accelerated endothelial progenitor cell growth (Figure 3) [162]. Salvianolic acid B (SAB), a traditional Chinese medicinal plant known to promote proliferation and migration of endothelial cells was combined with heparin to form a core solution in a PCL/collagen shell. A sustained release of 56% of SAB and 68% of heparin within the first 30 days was observed. In a rat subcutaneous embedding model, the biocompatibility of the engineered scaffold was confirmed, uncovering this strategy as promising for preventing acute thrombosis and for promoting rapid endothelialization [163].

### 4.5. Bone 

Bone is a mineralized connective tissue with functions of support and protection in the human body that, despite its inert appearance, is highly dynamic, being continuously resorbed by osteoclasts and neoformed by osteoblasts [164]. It is a very complex system with a large amount of extracellular matrix and a limited cell population [127]. A variety of materials have been used in the production of such biodegradable fibrous constructs for replacement and repair of damaged or traumatized bone tissues. Typically, engineering bone requires an artificial extracellular matrix or fibrous and highly porous scaffold, osteoblasts or cells that can differentiate into osteoblasts, and regulating factors or bioactive molecules to instigate cell recruitment, differentiation and mineralization to form new bone tissue [165,166].

**Figure 4:**
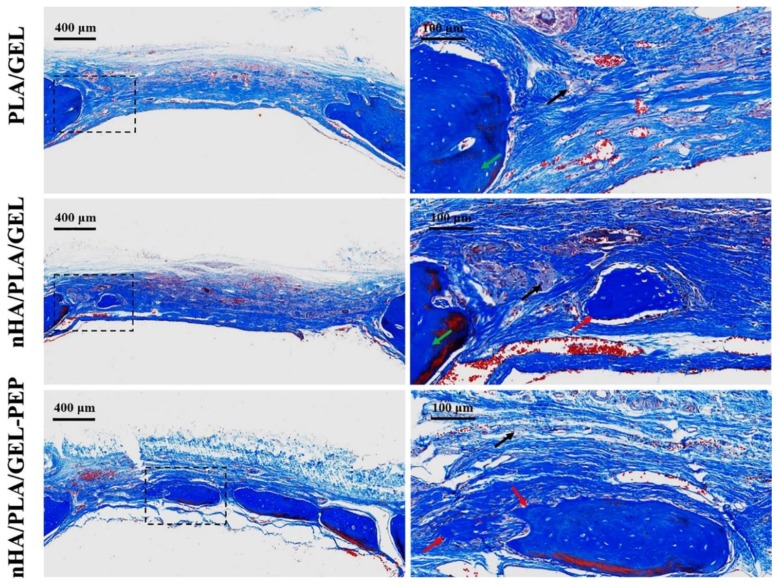
Masson’s trichrome-stained images of the newly formed bone within the repaired tissue eight weeks after surgery, using scaffolds made of PLA/gelatin (GEL), nano-hydroxyapatite (nHA)/PLA/GEL and nHA/PLA/GEL/BMP-2 peptide (-PEP). Red arrows indicate new bone, green arrows indicate host bone and black arrows indicate residual scaffolds. Residual scaffolds were all clearly filled with intercellular collagen fibers stained blue, and the newly formed bone tissue was dark blue because of the existence of abundant and compact collagen. New bone regenerated in the nHA/PLA/GEL-PEP group existed both in the middle and limbic of the defects (used with permission from [171]).

Lisignoli et al. developed a non-woven hyaluronic-acid-based polymer scaffold combined with bone marrow stromal cells and fibroblast growth factors and followed the osteogenesis of large segmental radius defects in rat models, up to 200 days of implantation. It was seen that bone mineralization was significantly induced by the presence of the growth factors, detected by the improved expression of important bone markers (i.e., alkaline phosphatase, bone sialoprotein, collagen type I, etc.). Further, new bone growth and lamellar bone percentage were highly correlated [167]. Mineralization and type I collagen production were also improved using microporous, non-woven, electrospun PCL scaffolds cultured with mesenchymal stem cells derived from the bone marrow of neonatal rats. After 4 weeks of culture, cells were seen to penetrate the scaffolds and to give rise to an abundant extracellular matrix [168]. Using an equal combination of scaffold and cells, Shin et al. supplemented these constructs with osteogenic factors using a rotating bioreactor and then implanted them in the omenta of rats for 4 weeks to assess new bone formation in vivo. It was observed that the constructs maintained their size, shape and bone-like appearance at the end of the 4 weeks and that cells and extracellular matrix covered the entire fibrous construct. Moreover, mineralization and type I collagen were also detected [169]. PCL has also been blended with PLA to produce nanofibrous, highly porous scaffolds with interconnected open porous structure with improved mechanical stiffness and bioactivity. Results showed that this combination not only enhanced cell viability of human mesenchymal stem cells but also promoted osteogenic differentiation. In addition, PCL/PLA scaffolds were seen to facilitate new bone formation in a critical-sized cranial bone defect mouse model [170]. Ye et al. engineered a scaffold of nano-hydroxyapatite/PLA/gelatin with a nanofibrous porous structure by combining homogenization, freeze-drying, and thermal treatment approaches. A derived peptide from the bone morphogenetic protein 2 (BMP-2-derived peptide) was then immobilized onto the surface of the fibrous scaffold via polydopamine. In vitro studies demonstrated the capacity of the altered scaffolds to instigate the alkaline phosphatase activity of bone mesenchymal stem cells and the gene expression related to osteogenic differentiation. In vivo examinations using a rat cranial bone defect model confirmed this scaffold ability to induce bone formation within the defects (Figure 4) [171]. Blends of PLLA and gelatin have been tested as well to produce fibrous scaffolds with β-cyclodextrin grafted with nano-hydroxyapatite and loaded with simvastatin, a known instigator of osteoblasts viability and differentiation. Data collected showed these nanostructured scaffolds to significantly increase the production of alkaline phosphatase, mineralization, osteogenic gene expression and bone regeneration, providing definitive proof of the potential of fibrous, porous biodegradable constructs for bone substitution/repair [172].

### 4.6. Cartilage

**Figure 5:**
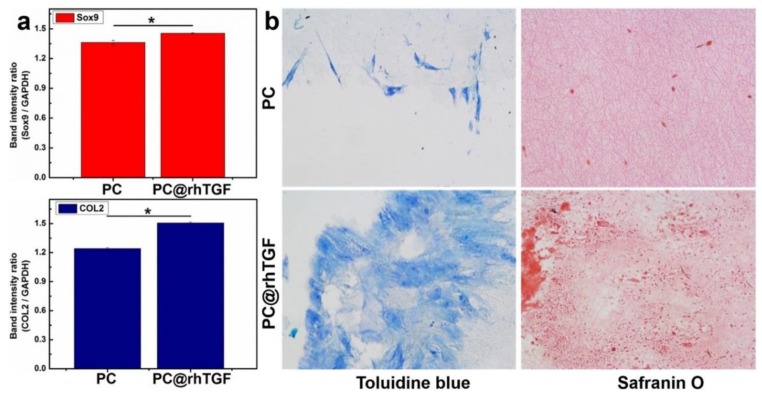
Chondrogenic differentiation of mesenchymal stem cells derived from Wharton’s jelly of human umbilical cord on two different nanofibrous scaffolds, the PLLACL and collagen, here referred to as PC; and the PLLACL, collagen and rhTGF-β3, here named PC@rhTGF. (**a**) Real time-qPCR analysis of chondrogenic markers SRY-box transcription factor 9 (So × 9) and collagen type II (COL2) after culturing for 14 days (*n* = 3, * *P* < 0.05). (**b**) Histological staining of glycosaminoglycans synthesized by the mesenchymal stem cells derived from Wharton’s jelly of human umbilical cord with Toluidine and Safranin O after culturing for 21 days (used with permission from [178]).

The articular cartilage can tolerate intensive and repetitive physical stress with great ease. However, it possesses a limited capacity to heal even the most minor injuries. This happens because of the reduced availability of chondrocytes, which are embedded in the dense extracellular matrix of the articular surface restricting their mobility, and absence of progenitor cells in the proximities of the wounded sites. In addition, the articular cartilage is an avascular, aneural, alymphatic tissue that only contains chondrocytes, which reduces its self-healing capacity [173]. The use of three-dimensional biodegradable scaffolds engineered with chondrocytes or progenitor cells has been seen, for many years, as an alternative to the limited success of the multiple surgical techniques available. In fact, Freed et al. demonstrated just that, by engineering cartilage implants from PGA fibrous scaffolds seeded with rabbit articular chondrocytes. These constructs were implanted as allografts on adult rabbits to repair knee joint defects. Cartilaginous repair was observed after six months of implantation. Compared to PGA alone, the cell-seeded PGA scaffolds improved the chondrocytes columnar alignment, the reconstitution of the subchondral plate, the spatial uniform distribution of glycosaminoglycans, and the bonding of the repair tissue to the underlying bone [174]. In a similar study, Li et al. evaluated the cell-seeded nanofibrous PCL scaffold regenerative properties in swine models. PCL constructs were seeded with allogeneic chondrocytes or xenogeneic human mesenchymal stem cells to repair iatrogenic, full-thickness cartilage defects during a six-month implantation period. In the end, the scaffolds seeded with mesenchymal stem cells were shown to regenerate hyaline cartilage-like tissue and to restore a smooth cartilage surface whilst maintaining the highest equilibrium of compressive stress, while the chondrocyte-seeded constructs produced mostly fibrocartilage-like tissue with a discontinuous superficial cartilage contour [175]. More recently, co-cultures of articular chondrocytes and mesenchymal stem cells were seeded onto electrospun PCL scaffolds with the purpose of repairing osteochondral defects in the trochlear groove of Lewis rats. After twelve weeks of implantation, hyaline-like cartilage tissue was found on the co-cultured scaffolds, while the bare PCL formed fibrocartilage, which cannot support the original cartilage function and deteriorates rapidly. It was also shown that both chondrocyte samples and co-cultures generated an equal level of cartilage repair, demonstrating their potential in vivo [176]. Still, the repaired tissue revealed inferior mechanical properties. Considering this, Kim et al. proposed the production of PCL/hyaluronic acid fibrous scaffolds loaded with transforming growth factor-β3 for cartilage repair of microfractures in a large animal model such as the minipig. After twelve weeks of implantation in vivo, the loaded scaffolds improved histological scores and increased type 2 collagen content and the overall mechanical performance [177]. Core-shell nanofibrous scaffolds were fabricated to encapsulate bovine serum albumin and the recombinant human transforming growth factor-β3 (rhTGF-β3) for tracheal cartilage regeneration. PLLACL was combined with collagen to generate the shell portion of the fibers. In vitro testing revealed that rhTGF-β3 could be released at a sustained and steady pattern without losing its biological activity. The rhTGF-β3-loaded scaffolds were seen to promote the chondrogenic differentiation of mesenchymal stems cells derived from Wharton’s jelly of human umbilical cord (Figure 5) [178]. Chitosan-based composite nanofibers containing graphene oxide (GO) have also been produced and their potential for cartilage regeneration evaluated. Due to its transport properties, allied with its flexibility and the one-atomic-thickness two-dimensional structure, GO has been pointed out as exhibiting very good biocompatibility because of the presence of abundant oxygen functionalities [179]. Tensile strength experiments revealed that the incorporation of GO increased the mechanical properties of nanofibers. At concentrations of 6 wt%, GO appears to generate a highly biocompatible environment conducive with ATDC5 cells (an excellent in vitro cell line model for skeletal development) proliferation [180]. Surface modification of gelatin can provide a new generation of biopolymers and fibrous biotextiles with chemical, mechanical and biological properties desirable for cartilage tissue engineering. Agheb et al. proposed functionalizing gelatin with tyrosine protein and 1,2,3-triazole ring. In vitro cell culture studies demonstrated the electrospun engineered protein scaffold to support attachment and growth of cells while maintaining their viability. The results also showed that cross-linked nanofibers could be considered excellent matrices for chondrocyte adhesion and proliferation in cartilage tissue-engineering applications [181]. The former in vitro and in vivo studies verified that spinning strategies may allow novel layered scaffolds to be constructed that simultaneously and effectively deliver growth factors and fulfill cell migration in a controlled manner to promote tissue regeneration.

### 4.7. Ligament

Ligaments are short bands made of strong, flexible, dense connective fibrous tissue found in between bones and responsible for joint movement, stability and load transfer. They are mainly formed of oriented bundles of collagen fibers. Depending on our daily activities, the ligaments of some joints may be subjected to greater strain than others, making them more prone to injury. It is clear that the ligaments from knees or legs are subjected to added strain due to body weight and may suffer more serious consequences. In fact, one of the most common ligament ruptures occurs at the anterior cruciate ligament (ACL), which connects the femur to the tibia and acts as a main stabilizer of the knee [182]. Rupture of the ACL results in abnormal joint kinematics and often leads to irreversible damage, as it does not heal naturally and requires surgical intervention [183]. Spinning techniques associated with tissue engineering tools offer new alternatives for ACL treatment by means of biodegradable, fibrous scaffolds with or without seeded cells. The ideal scaffold must provide high mechanical strength at the initial moments of implantation and gradually lose it, degrading as new tissue is formed [184].

Ouyang et al. prepared seven different biodegradable scaffolds, using a solvent spin-casting technique, from PCL, PDLA, PLLA, PLGA and mixtures of PLA/PCL and studied the adhesion, proliferation and morphology of rabbit ACL cells and bone marrow stromal cells. Data revealed that high-molecular-weight PLGA scaffolds were more likely to allow cells to attach and proliferate and to promote cell expansion [185]. On the contrary, Lu et al., using the same polymers PGA, PLLA and PLGA engineered as three-dimensional braided, fibrous, interconnected scaffolds, established PLLA as the most suitable substrate (between the tested samples) for ACL tissue engineering. They observed the PLLA scaffolds pre-treated with fibronectin to maintain their structural integrity and mechanical performance over time. Primary rabbit ACL cells were also able to attach efficiently on these substrates and, thus, enhance the long-term matrix production [186]. Recent studies have also shown electrospun PCL scaffolds as good candidates for in vivo ACL reconstruction of a rodent model. After two, six and twelve weeks of implantation, Petrigliano et al. demonstrated gradual infiltration of collagen in both the bone tunnel and intra-articular regions of the scaffold, together with the increase in failure load and stiffness over time [187]. In turn, Leong et al., by incorporating fibroblast growth factors and human foreskin fibroblasts within the PCL grafts matrix, provided evidence of the fibrous constructs’ excellent healing and regenerative potential. Indeed, after 16 weeks of implantation on athymic rat models, infiltration of the grafts with cells and aligned collagen deposition with minimal inflammatory reaction were observed. Here too, the mechanical performance was improved over time [188]. PLCL and silk fibroin have been processed by electrospinning in three ways—random nanofibers, aligned nanofibers and aligned nanoarrays—and studied for their mechanical performance in light of ligament requirements. The Young’s modulus of the aligned nanoarrays was inferior to the other configurations; however, it provided larger pores and enough space for cell infiltration, which yielded improved cell proliferation for up to 28 days of culture. The aligned nanoarrays achieved a balance between porosity and mechanical properties highly desirable in tissue engineering [189].

For many years, the natural choice for ACL reconstruction fell on collagen-based fiber scaffolds. However, their mechanical strength and degradation rates were not easily controlled [190]. Lately, silk and collagen blends have been prepared to mimic the components of the ligament and thereby accelerate regeneration. Implantation in rabbits demonstrated that cell infiltration increased over time on these constructs and that more fibroblast-like cells were found in their core. Compared to autografts, silk/collagen fibrous scaffolds enhanced the most tendon-bone healing and instigated trabecular bone growth into the scaffold. In the end, their potential for clinical applications was established [191]. A hybrid scaffold composed of degummed knitted silk microfibers coated with bioactive basic fibroblast growth factor (bFGF)-releasing electrospun PLGA fibers was produced by Sahoo et al. In vitro testing demonstrated the ability of rabbit bone marrow mesenchymal stem cells to grow on these scaffolds and the bFGF to stimulate cell proliferation and gene expression, increasing collagen production and, hence, the fibrous construct mechanical properties [192].

## 5. Drug Delivery Systems

In the quest for efficient drug delivery systems, by means of systemic and topical routes, biotextile structures have been fashioned in a variety of formats, resembling interconnective tissues, the extracellular matrix and even organs. In fact, over the last fifty years, potential administration routes via biotextiles have gone from wounds, burns and dermatosis, the most common, to systemic diseases, implantable devices and nanoencapsulation [193]. Drug delivery systems based on biotextiles can now sustain local release, control deliverance, target cell/microorganism strains or biomolecules, and even boost smart release via local stimuli by means of topical, transdermal and implantable administration. In recent years, electrospun fibers designed to entrap biologically active molecules have proven efficient in protecting affected areas against pathogens this way, preventing microbial colonization and accelerating healing. The main requirement for a drug delivery system to be considered as an effective tool is its capacity to maintain the pharmacologically effective therapeutic drug levels for prolonged periods of time while still allowing “dosing-on demand” [194]. Innovative manufacturing techniques are emerging to design fibrous scaffolds with proper textured fiber meshes to achieve specific performances in different biomedical environments. Indeed, different strategies have been optimized to incorporate molecular species into polymer-based solutions either by direct (e.g., co-axial spinning) or indirect (e.g., co-spinning) encapsulation [1,195]. Drug-release profiles are mainly dependent on the physical–chemical properties of the polymer. As such, hybrid architectures made of natural and synthetic materials are also being engineered with the purpose of offering delivery platforms with controlled degradation rates, responsive to local stimuli, for targeted biomolecule release. Examples of biotextile-based drug delivery systems are explored in the following sections.

### 5.1. Topical

Topical administration of specialized therapeutic agents, such as antibiotics, plant extracts, antimicrobial peptides or proteins and nanoparticles, is a favored route for a localized action. This strategy is preferred in many cutaneous, eye and vaginal disorders because of its convenience, easy application, affordability, minimum toxicity, superior physiological and pharmacological responses, enhanced drug bioavailability and, since it requires non-invasive procedures, painlessness for the users. Topical systems are highly desirable as potential substitutes for systemically administered drug therapies, by minimizing side effects associated with drug dosage and off-target action. The most challenging aspect of designing such a therapeutic system relates to the delivery platform from which an optimal biomolecule concentration can remain active while being delivered for an appropriate time period [196,197]. Even though they are not as challenging as in systemic routes, topical administration still must overcome barriers that may limit the bioavailability of drugs and its deliverance at the effective site. Local infections masking the affected area are known to inhibit the action of therapeutic agents used to stimulate anti-inflammatory responses or cell growth and proliferation [198].

Nanofibrous biotextiles have been described to possess a large surface area capable of efficiently binding and delivering hydrophilic and hydrophobic drugs. Furthermore, their release rate can be tuned to meet the specific clinical demands of the affected area by modulating the fibers’ diameter, morphology, structural organization, porosity, drug protection (direct or indirect loading, namely co-axial or co-spinning) and content ratio between polymers and drug. Biomolecules can also be functionalized at the surface of the nanofibers by physical or covalent binding for a faster delivery [39].

Highly infected and chronic wounds are some of the clinical pathologies where topical administration of drugs via biotextile platforms are the most frequent and viable. Here, topical delivery vehicles can provide therapeutic action directly to the wound bed or the affected area, using one- or multiple-agent systems, potentially reducing unwanted side effects. Dual release electrospun scaffolds containing an anesthetic, lidocaine, and an antibiotic, mupirocin, were produced from PLLA using a dual-spinneret electrospinning apparatus. Their release rate was followed, revealing discrepancies between biomolecules: while lidocaine displayed an initial burst of 80% of its weight in the first hour, mupirocin only released 5%, therefore experiencing a more sustained release for effective antibacterial action. By comparing with single-spinneret electrospun mats, drug release kinetics was seen to alter due to the competitive behavior between molecules and the different polymer interactions generated [199]. Topical administration of levothyroxine, a synthetic hormone that stimulates lipid metabolism and induces lipolysis, has been reported to reduce deposits of adipose tissue in the skin. At high concentrations, however, potential systemic effects may be unleashed. To overcome this, nanofibrous textiles of blends of PVA and poly-N-isopropylacrylamide (PNIPAM) were proposed for sustained topical delivery of this hormone. Data reported the ability of the polymeric biotextile to sustain the release and penetration of levothyroxine within the skin while maintaining its effectiveness for longer periods and minimizing systemic adsorption [200]. PVA has also been combined with poly(vinyl acetate) (PVAc) to form nanofibers loaded with ciprofloxacin hydrochloride (CipHCl), a quinolone antibiotic used to treat a variety of bacterial infections. Here, too, both the kind of polymer and the amount of drug loaded greatly affected the degree of swelling, weight loss, and initial burst and rate of drug release. Pristine PVAc fibers were capable of sustaining release of 50% of the drug for 80 days, whereas the PVA nanofiber mats released the drug within 3 days. Blending the two polymers allowed for a convenient rate and period of drug release to be attained, optimal to fight skin infections [201]. Antimicrobial fusidic-acid-loaded electrospun PLGA ultrafine fibers have also been examined for their potential in treating infected wounds under dynamic conditions. It was seen that the engineered constructs allowed a progressively faster release of bioactive fusidic acid, eradicating planktonic bacteria and considerably suppressing biofilm formation. However, findings point out the risk of wound reinfection and microbial resistance from using non-medicated or inadequately medicated bioresorbable fibrous dressings [202]. The same recommendations were made by Alhusein et al. while reporting the release of tetracycline hydrochloride (tetHCl) from a triple-layered electrospun matrix, made of poly(ethylene-co-vinyl acetate) (PEVA) at the center, sandwiched between two layers of PCL [203]. The engineered topical scaffold was found very effective in entrapping the antibiotic and promoting their efficient timely local release, inhibiting the growth of a panel of bacteria, including clinical isolates. PCL and hyaluronic acid have been conjugated with epidermal growth factors via emulsion electrospinning, revealing significant synergistic effects that contributed to cell proliferation and infiltration, ultimately leading to an enhanced regeneration that culminated in fully functional skin. Moreover, an up-regulation of wound-healing-related genes like collagen I, collagen III and TGF-β was also reported [204]. Even though blend emulsion electrospinning has been an option in many studies, there are others that defend this strategy to destroy the bioactivity of proteins or to make the electrospun dope highly unstable [205]. Using bovine serum albumin as a carrier protein, Peh et al. proposed a simultaneous blend-spun of Vitamin C, Vitamin D3, steroid hormone hydrocortisone, insulin, thyroid hormone triiodothyronine, and epidermal growth factors into PLGA-collagen nanofibers. The engineered strategy allowed for a target-release of each biomolecule, with Vitamin C facilitating collagen I secretion by fibroblasts, insulin potentiating adipogenic differentiation and Vitamin D3, steroid hormone hydrocortisone, insulin, thyroid hormone triiodothyronine and the epidermal growth factors stimulating skin fibroblast and keratinocytes proliferation [206]. Aside from infected wounds, topical skin administration of biomolecules has also been employed in the treatment of other disorders, namely keloids. These are fibroproliferative lesions that occur at areas of cutaneous injury. They are benign but often cause pain, tenderness, pruritus and paresthesias. Li et al. proposed a new strategy to treat these conditions by co-delivering dexamethasone and green tea polyphenols at the affected area using PLGA nanofibers. The engineered biotextile was characterized as multi-functional by including capacities to maintain a moist environment, resisting bacterial infection and controlling drug release. After a three-month period of treatment, these PLGA/dexamethasone/green tea polyphenols fiber meshes were found to significantly increase the degradation of collagen fibers in keloids compared to the traditional methods [207]. Another benign skin condition of long duration that affects most people during adolescence is acne. Current strategies to treat acne resort to antibiotics and biomolecules delivered to the skin in the form of pills, ointments, gels or soaps. Most recently, Khoshbakht et al. proposed the fabrication of tretinoin-loaded PCL nanofibrous mats as a potential anti-acne patch. Electrospun nanofibers showed a prolonged release of tretinoin, which was then reflected in a superior antibacterial action. Additionally, the drug-loaded construct showed inherent stability under various storage conditions at room and fridge-preserving temperatures, anticipating that the easy fabrication, low costs and dosing frequency of this strategy may offer a new therapeutic platform for treating acne disorders [208].

Electrospun nanofibers have also been applied in eye diseases. One of the most challenging issues in treating eye diseases is the very short residence time of the drug. Most drugs are eliminated within a few seconds due to the poor capability of the eye to accommodate additional liquids. As such, alternative drug delivery systems have been engineered in the form of fibers and gels. Voriconazole, a triazole antifungal agent with low aqueous solubility, good oral bioavailability, acceptable tolerability and promising activity against resistant fungal species and fungal isolates associated with keratitis, has been incorporated into polyvinyl alcohol (PVA)/hydroxypropyl-β-cyclodextrin (HPβCD)-blended nanofibers for efficient ophthalmic delivery. Drug loading was significantly enhanced by the presence of HPβCD. Compared with a voriconazole free state solution, the nanofibers significantly prolonged the antibiotic half-life and increased its bioavailability in rabbit tears. Further, no obvious signs of irritation were detected after application in the conjunctival sac [209]. Dendrimer-based nanofibers made of polyamidoamine have also been examined as topical delivery vehicles for the glaucoma drug brimonidine tartrate. These systems were considered non-toxic and did not cause ocular irritation in animal tests using a normotensive rat model. Intraocular pressure response also improved with daily dosing [210]. Recently, Göttel et al. proposed a system based on gellan gum/pullulan electrospun nanofibers shaped into curved geometries and capable of turning into a gel upon administration. A clear prolongation of the fluorescein residence time compared to conventional eye drops was confirmed using the developed in situ gelling system [211]. Grimaudo et al. also showed the ability of hyaluronic acid nanofibers to work as a dual delivery system for ferulic acid, an antioxidant, anti-aging, anti-inflammatory, neuroprotective and hemato-protective agent, and ε-polylysine, a water-soluble, biodegradable antimicrobial peptide active against bacteria, fungi and yeast. The engineered multi-action system demonstrated a controlled release of the biomolecules within the first hour of contact without inducing a cytotoxic response, and with an effective action against relevant microbial species prevalent in chronic ocular diseases [212]. Once again, the value is proven of nanofiber-based delivery systems as alternatives to aqueous drug-based solutions to treat ocular-related disorders. Still, topical formulations are less effective in treating retinal inflammatory diseases. As such, Singla et al. proposed the development of preservative-free fluocinolone acetonide-loaded PCL nanofibers. This corticosteroid is used in dermatology to reduce inflammation and relieve itching. Here, both plasma and ocular kinetics supported the therapeutic utility and target-site deliverance of the fluocinolone acetonide, without systemic distribution. This single application maintains the therapeutic window for longer periods, thus ensuring higher patient adherence and compliance. PCL was once again highlighted as a promising drug carrier. Apart from the already-mentioned features, PCL gained popularity as a potential alternative for human amniotic membrane by promoting adhesion, supporting cell proliferation and infiltration to form a three-dimensional corneal epithelium [213].

A great number of vaginal disorders arise from sexually transmitted infections. For that reason, multipurpose prevention technologies (MPTs) that simultaneously prevent sexual diseases and unintended pregnancy remain a global health priority. Combining chemical and physical barriers in one potential therapeutic formulation has been a great challenge. Ball et al., using FDA-approved polymers, polyethylene oxide (PEO) and PLLA, produced nanofiber meshes with tunable fiber size and controlled degradation kinetics to enable the topical release of multiple agents against HIV-1, HSV-2, and sperm at the vaginal mucosa. Data reported the capacity of these electrospun biotextiles to inhibit HIV-1 infection and to physically obstruct sperm penetration, providing a physical coverage for both the vaginal epithelium and cervix. Here, multiple non-hormonal chemical contraceptive alternatives were screened, uncovering the potential of glycerol monolaurate to inhibit sperm motility and viability in a dose-dependent manner [214]. Formulations to co-deliver more than one therapeutic agent in vaginal disorders or as contraceptive agents are very frequent. Biotextiles made of PVA-based nanofibers have been fashioned and loaded with tenofovir and levonorgestrel (a contraceptive progestin) using a production-scale electrospinning equipment. The engineered fabrics showed good drug association efficiencies and a reasonably high drug loading, the release rate of which could be modulated by changing the fiber architecture’s polymer/drug ratio. In vitro studies demonstrated this system low cytotoxicity and the ability to sustain the anti-HIV activity of tenofovir [215]. Huang et al. formulated a therapy in which the polymeric drug delivery platform would release specialized antiretroviral drugs responsible for fighting sexually transmitted diseases only upon contact with sperm. Cellulose acetate phthalate (CAP)-based nanofibers were produced by electrospinning and loaded with etravirine and tenofovir disoproxil fumarate agents. Because of the CAP pH sensitivity, fibers were insoluble in simulated vaginal fluid at pH 4.2 but dissolved quickly upon contact with human semen at pH 7.0–8.5, thus releasing the drugs. CAP also possesses anti-HIV activity, which may include additional effects to this therapeutic topical strategy [216]. Current approaches also include the combination of nanofiber biotextiles functionalized with nanocarriers containing therapeutic biomolecules. These strategies have been recently proposed due to the poor retention and extensive leakage of nanocarriers (in the form of nanoparticles or nanocapsules) in topical vaginal administration. Such an example is the research of Krogstad et al., in which a nanoparticle-releasing nanofiber delivery platform was developed by combining mucoadhesive fibers for better retention in the vaginal tract, and PEGylated nanoparticles loaded with etravirine, a topical microbicide for HIV prevention, that diffused quickly through the mucus. This composite formulation was seen to provide 30-fold greater retention of nanoparticles in the reproductive tract compared to aqueous suspensions. Further, the functionalized nanoparticles displayed sustained and higher etravirine concentrations up to 7 days of culture, demonstrating the efficacy of single-dose topical therapies [217].

### 5.2. Transdermal

Transdermal applications of bioactive dressings aim at transporting drugs into the bloodstream by penetrating the skin barrier. In the past, only a few drugs were approved as transdermal patches, like steroid hormone and nicotine, due to their bioavailability [218,219]. In recent years, there has been an increased interest in transdermal delivery systems as a new approach for drug administration. Contrary to systemic products, transdermal patches can provide a sustained release of a drug from the device into the skin, are non-invasive systems, avoid gastric irritation and bypassing the first-pass metabolism, and are relatively low toxic. Another benefit of using biotextile transdermal delivery systems is the ability to very easily and quickly stop a therapeutic treatment in case of adverse effects [220,221].

Different transdermal delivery systems have been designed following the evolution of polymer science [222]. Natural, synthetic and hybrid polymers are the backbone of transdermal delivery systems as they allow to control the release of drugs through the intact skin [223]. Frequently, polymers are processed in the form of nanofibers and used as delivery platforms to treat difficult wounds, both mechanically and chemically [224]. Aside from entrapping bioactive molecules necessary to induce healing or fight infections, they also absorb excess exudates and facilitate oxygen permeability via their large surface area and interconnected open pore structure, respectively. Further, the nanofibrous constructs morphology and chemical composition may also condition the loading capacity and release profile of the drugs. Currently, there are even smart strategies that release drugs in response to the environment ion or enzyme concentrations and pH levels [221].

The absence of several types of vitamins in the human body can lead to severe health problems ranging from megaloblastic anemia to Parkinson’s disease [225,226]. In an attempt to improve transdermal delivery of Vitamin B12, Vitamin E and Vitamin A and surpassing more effectively the skin barrier, Madhaiyan et al. [219] and Taepaiboon et al. [227] explored alternative routes based on nanofibrous constructs made of PCL and cellulose acetate, respectively. Vitamin B12 (cobalamin) is a water-soluble vitamin necessary for red blood cell formation, neurological function and DNA synthesis and is naturally present in some foods [226]. In turn, Vitamin E and Vitamin A acid are lipid-soluble substances that prevent skin disorders and have antioxidant properties [227]. Vitamin B12-loaded PCL electrospun fibers were surface-modified by plasma treatment to increase the period of fiber degradation, loading capability and hydrophilicity, thereby enhancing the vitamin release rate above untreated PCL. The plasma-treated PCL transdermal patch allowed a release of 34% of vitamin B12 per day, equivalent to 340 mcgs, the daily requirement in cases of vitamin deficiency. The increased hydrophilic nature of the patch facilitated release because of water-sorption-mediated drug desorption [219]. Similarly, mats made of electrospun cellulose acetate loaded with vitamin E (5 wt%) and vitamin A acid (0, 5 wt%) were produced by eletrospinning. As expected, due to the low stability of Vitamin A acid, cellulose acetate fibers could incorporate more Vitamin E (≈83%) than Vitamin A acid (≈45%). Still, in both cases, their encapsulation efficiency was confirmed. Subsequent studies of drug releasing in a acetate buffer solution showed that fibers were stable and capable of maximum release of Vitamin E and Vitamin A acid within 24 and 6 h, respectively, highlighting this biotextile as an effective transdermal drug delivery system [227].

The local delivery of drugs is preferred to systematic administration in wounds as it accelerates healing by speeding the healing phases in acute injuries and by administrating more effectively antimicrobial agents capable of preventing or fighting infections. Drug-loaded biotextiles containing antibiotics, anti-inflammatory substances and biomolecules are an excellent alternative to produce functionalized biological and biochemical dressings for wound therapies. Both synthetic and natural-origin polymers, like PVA and chitosan, combined with antibiotics or biomolecules have shown great performance in accelerating the rate of healing and controlling drug release, making them suitable for transdermal drug systems [228,229]. Kataria et al. produced biodegradable PVA and sodium alginate modified by active loading with ciprofloxacin, a fluroquinolone antibiotic (agents commonly applied against microorganisms present in skin infections) to prevent wound infections. Data reported the nanofiber transdermal patch to follow the Higuchi and Korsmeyer–Peppas model for drug release, with a controlled, sustained delivery with time. Once again, loaded patches were more effective in controlling infections, accelerating healing and promoting re-epithelialization (in vivo) than the free state antibiotics or unloaded patches [228]. The same was reported by Mendes et al. when mixing chitosan with phospholipids to generate electrospun hybrid nanofibers and posteriorly loading the structure with a variety of substances, including curcumin (an antioxidant, anti-inflammatory agent and an inhibitor of tumorigenesis and metastasis), diclofenac (anti-inflammatory agent) and Vitamin B12. Here too, the hybrid biotextile presented great stability in physiological environment due to chemical interactions established between the natural polymer and the biomolecules, suitable biocompatibility and a controlled, sustained release of all tested substances [229].

**Figure 6:**
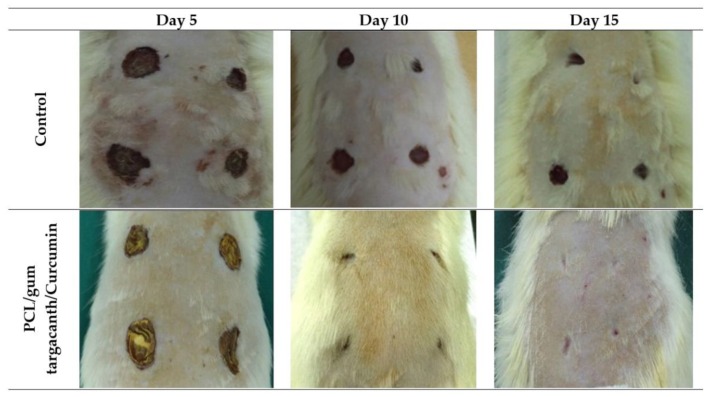
Evidence of accelerated wound closure in diabetic rat models (in vivo testing) promoted by curcumin-loaded biotextiles (adapted with permission from [230]).

Curcumin is a naturally occurring poly-phenolic compound with innate antimicrobial action and a broad range of biological functions, including anticancer, antioxidant, anti-infective, angiogenic and anti-inflammatory activities. However, its in vivo low bioavailability and fragile stability demands suitable carrier vehicles to be used to sustain its action and continuous release towards affected areas. In that sense, Ranjbar-Mohammadi et al., by producing curcumin-loaded PCL/gum targacanth nanofibers for wound healing disclosed their ability for applications in diabetic conditions. Indeed, after 15 days of culture, pathological studies demonstrated the markedly fast wound closure promoted by the engineered biotextiles, with well-formed granulation tissue dominated by fibroblast proliferation, collagen deposition, complete early regenerated epithelial layer and formation of sweat glands and hair follicles (Figure 6) [230]. Ravikumar et al., using a CAP nonwoven template, achieved similar results, confirming the liberation of curcumin in a controlled manner via transdermal delivery [231]. The same was observed by Rramaswamy et al. using tetrahydro curcumin loaded onto PCL/PEG hybrid formulations processed in the form of electrospun hybridized transdermal patches. They also concluded that drug diffusion in such systems follows Higuchi’s model diffusion mechanism [232].

In recent years, the world population has been facing a dominant problem that prevails in most developed countries and affects millions of people, namely obesity [233]. Ariamoghaddam et al., using a blend of PVA/gelatin loaded with curcumin, verified this natural extract as a potent anti-obesity agent and engineered a transdermal delivery patch to decrease the sub-cutaneous volume of adipose tissue in obese rats. Curcumin-loaded patches were applied in the abdomen region of obese rats for 6 weeks. Afterward, the rats were exposed to magnetic resonance imaging and compared to control groups consisting of normal-diet and high-calorie-diet rats. Results confirmed that these modified PVA/gelatin-based biotextiles effectively deliver curcumin biomolecules through the skin and that these patches were efficient in decreasing the volume of adipose tissue within the tested subjects by 4% to 7%. Once again, the capacity of bio-synthetic hybrid polymers was demonstrated to generate sustainable drug-loading nanofiber systems, with optimal transdermal delivery, as alternatives to conventional systemic therapies [234].

### 5.3. Implantable

Implantable drug delivery systems are designed to store and deliver small, precise doses of therapeutic drugs or medicines into the bloodstream or directly to specific tissue sites. The major advantages of these systems include targeted local delivery of the drug at a constant and predetermined rate, thereby minimizing dose required and potential side effects while improving therapeutic efficacy [194].

Implantable drug delivery systems essentially consist of a micropump that contains a reservoir in which the pharmaceutical drug in gaseous or liquid form is stored, an actuator release or pump mechanism, inlet and outlet valves, and in some cases a catheter to directly deliver the drug to a target site. Alternatively, the drug can be blended with the implantable materials and then, by using compression- or injection-molding techniques, generate a device with a pre-defined architecture. However, controlling of the structure and the internal architecture of the system has proven a real challenge. Further, in many instances, the drug becomes inaccessible or loses its activity. Biotextiles have been proposed as novel strategies for the development of implantable drug delivery systems with applications in long-term diseases, including cardiovascular, tuberculosis, diabetes, cystic fibrosis, glaucoma, cancer, etc. [235]. Indeed, in Section 4, some of those implantable systems have already been analyzed and examples of bioactive molecules or antibiotics released from biotextiles have been explored. Still, one of the most important applications of fibrous implantable drug delivery systems remains to be disclosed, namely the detection and treatment of cancer.

Polymer-based systems have raised much attention in the last few decades as a means of achieving high therapeutic concentrations of chemotherapy to the site of malignant diseases in cancer patients by implanting drug-loaded systems intra-tumorally or in areas adjacent to the cancerous tissue. Most of these devices are fashioned from biodegradable polymers to circumvent a second surgery for device removal, in already debilitated patients, and to avoid a chronic foreign-body immune response. The development of such devices is guided by the desire to improve overall survival and quality of life by patients [236].

Gao et al. proposed the incorporation of 5-fluorouracil, a hydrophilic anticancer drug more effective when administered at lower doses for longer periods, onto PLLA fibers. The fabrication process selected was wet-spinning. The drug release rate was regulated by optimizing the processing parameters, such as drug content, polymer concentration, nonsolvent composition and extrusion flow rate. Most of the drug was encapsulated into the PLLA bulk fibers, achieving good-term release profiles with small initial burst, desired for cancer treatments [237]. This drug, together with oxaliplatin, is currently a mainstay of adjuvant chemotherapy in colorectal cancer patients. However, systemic delivery exposes not just tumor cells but other body organs to their toxicity. Moreover, oxaliplatin-induced peripheral neuropathy remains a main concern to the use of this drug. As such, these two drugs have been loaded onto PLLA electrospun nanofibers and their effects examined when directly exposed to human colorectal cancer HCT8 cells (in vitro) and colorectal CT26 tumor-bearing mice (in vivo). In both situations, the drug-loaded PLLA fibers displayed antitumor efficacy in a time-dependent manner, sustaining drug release for longer, and suppression of tumor growth, which prolonged the animal’s survival [238]. Oxaliplatin has also been tested in combination with dichloroacetate, a metabolic modulator, using a dual drug-loaded multilayered PLLA system. This strategy was proven to be effective in sustaining the local release and action of the drugs in a time-programmed manner. Moreover, the synergistic effect between the two drugs was demonstrated to prevent local cancer recurrence following surgery [239].

Doxorubicin (DOX) is an anthracycline antibiotic with antineoplastic activity, commonly applied as chemotherapy medication to treat a variety of cancers (breast, bladder, lymphomas, leukemia, etc.). Systemic administration of this drug is associated with severe toxicity in healthy tissues, limited distribution, low resection rates and, overall, poor patient survival. To overcome these limitations, Yang et al. developed a localized, implantable drug delivery device made from hydrophobic DOX-encapsulated active-targeting micelles assembled from a folate-conjugated PCL-PEG copolymer. These micelles were then incorporated in a PVA matrix, forming the core of co-axial electrospun nanofibers surrounded by gelatin. The engineered nanocarrier delivering system was seen to reduce drug dose requirements, frequency of administration and chemotherapeutic-related side effects while maintaining an effective therapeutic action against artificial solid tumors [240]. Similar observations were made by loading DOX onto mesoporous silica (MSNs) nanocarriers and then by incorporating those structures into PLLA nanofibers. Loaded scaffolds were successfully fashioned, exhibiting good nanocarrier distribution and improved thermal stability. More importantly, they allowed for high doses of DOX to be loaded, sustaining its release for longer periods than MSNs- or DOX-free counterparts, thus increasing their in vitro antitumor efficacy without compromising the viability of surrounding healthy cells [241]. In a following study, Liu et al. produced a biotextile in which the DOX was loaded directly onto PLLA without using a ceramic nanocarrier. Secondary hepatic carcinoma mice models were prepared by injecting murine mammary carcinoma EMT6 cells into the left hepatic lobe and into the portal vein of Balb/c mice. DOX-loaded PLLA mats were then used to cover the affected areas. The growth of the nodular secondary hepatic carcinomas was significantly inhibited by the prolonged, controlled release of DOX, whereas the median survival time of the mice bearing diffuse secondary hepatic carcinomas was increased from 14 to 38 days. Throughout that time, neither injury to neighboring liver tissues nor systemic adverse reactions were observed [242]. To establish an even greater optimal control of drug targeting, Li et al. proposed the use of DOX-loaded photoluminescent MSNs modified with a pH-sensitive polydopamine “gatekeeper” for quick release and faster uptake by cancer cells. These photoluminescent nanocarriers were functionalized at the surface of PCL/gelatin electrospun nanofibers modified with photothermal carbon nanoparticles, forming a highly specialized implantable biotextile. Compared to drug administrations in the free form, the implant was seen to significantly enable a superior cell uptake effect, thus increasing the drug efficacy against tumor cells by responding to under-near-infrared irradiation. The photothermal effect of the carbon nanotubes weakened the electrostatic interaction between the photoluminescent nanocarriers and the PCL/gelatin nanofibers, resulting in the controlled release and, subsequently, internalization of DOX for a more effective cancer cell killing action [243].

## 6. Conclusions and Future Perspectives

The ideal design for tissue engineering devices falls within the features of fibrous constructs obtained via spinning techniques. It is generally agreed that biocompatible, biostable, biodegradable, porous devices are the most appropriate for both hard and soft tissue repair and substitution. They are designed with a fiber-based, highly porous and interconnected architecture that resembles the extracellular matrix, creating in this way an environment conducive with cell penetration, adhesion, proliferation and ultimately tissue regeneration. In many cases, the association with biomolecules, such as drugs, plant extracts, growth factors, proteins, peptides or essential oils may facilitate this task, giving rise to controlled biomolecule delivery systems that not only promote tissue regeneration but fight infections as well.

Among the spinning techniques available for fibrous architecture production for tissue engineering or biomolecule delivery applications, electrospinning can be highlighted by the ease of process but most importantly by the ability to control in great detail each processing parameter to obtain large surface area fibrous structures at the nanometer scale. Biodegradable polymers have gained an important status over the years by replacing biostable temporary therapeutic devices, used only for substitution, with systems capable of stimulating the body to repair and regenerate while degrading at an equal rate. These biodegradable fiber-based constructs are fashioned with desirable mechanical strength, structural integrity, large surface area and open pore structure to successfully respond to local demands. Loading with biomolecules may ease this process and make the integration of the antimicrobial biomaterials quicker and less harmful for the patient.

Nowadays, biotextiles developed from spinning techniques are in great demand. They combine textile technologies with antimicrobial biomaterials to generate vascularized therapeutic devices for a variety of applications. Indeed, there are surgical meshes, wound dressings, ligaments and soft tissue substitutes currently being tested in clinical trials that are based on the concepts revised in the present review. The main challenges related to the application of biotextiles in tissue engineering consist of the combination of textile machinery with biomaterials and the advances necessary to generate tissues and organs automatically. For instance, the manufacturing process of fibers developed from synthetic materials hampers the capacity of cell encapsulation inside the fibers, establishing the need for more advanced fiber fabrication techniques; therefore, it is likely that the field of tissue engineering will advance towards that direction. Another obstacle relies on the inability of implantable fabrics to capture the in vivo mechanic and biological properties of the organs and tissues. Indeed, clinical applications of such constructs have demonstrated that in vitro and ex vivo analysis results in quite different outcomes than actual real-life conditions. An alternative to address this challenge would require the use of fibers from biomaterials with adjustable properties that enable the delivery of growth factors or by the application of textile-based tissues on the development of in vivo disease models and drug testing platforms. Furthermore, new smart fiber-forming polymers are being developed with unique properties, and as soon as they are spun, they may function as biosensors, actuators and drug delivery systems responsive to pH, temperature and drug concentration. Moreover, with computational capabilities advances, computational approaches can be used to better understand the resorption properties and mechanisms by controlling the processing parameters and the different chemical compositions.

## Figures and Tables

**Table 1:** Synthetic biodegradable polymers used in tissue engineering: Physical, mechanical and degradation properties.

Polymer	Melting Point (°C)	Glass Transition Temperature (°C)	Tensile Modulus (Gpa)	Elongation (%)	Degradation Time (months)	Reference
Polycaprolactone (PCL)	58–63	(−65)–(−60)	0.2–0.4	300–1000	>24	[19]
Poly(glycolic acid) (PGA)	220–233	35–40	6.0–7.0	1.5–20	6–12	[19]
Poly(lactic-co-glycolic acid) (PLGA)	Amorphous	45–55	1.4–2.8	3–10	1–12 (adjustable)	[20]
Poly(lactic acid) (PLA)	150–162	45–60	0.4–3.5	2.5–6	>24	[21]
Poly (L/D-lactide) (PLLA or PDLA)	170–200	55–65	2.7–4.1	3–10	>24	[21]
Poly (DL-lactide) (PDLLA)	Amorphous	50–60	1–3.5	2–10	12–16	[21]
Polydioxanone (PDO)	N/A	−10–0	1.5	N/A	6–12	[19]

**Table 2:** Some of the representative spinning systems studied for biomedical applications.

Polymeric Matrix	Processing Method	Bio-Application	Reference
PLA/CNW	Melt-spinning	-	[83]
PHBV/PLA	Melt-spinning	Textile implants	[82]
PLGA	Dry/wet and Wet-spinning	Scaffolds production	[84]
CS	Dry-spinning	Tissue regeneration	[85]
PCL	Wet-spinning	Regeneration of smooth muscle cells	[86]
GN	Wet-spinning	Tissue regeneration	[87]
GN/SA	Wet-spinning	Enzyme immobilization	[88]
PCL	Wet-spinning	Regeneration of smooth muscle cells	[86]
Collagen	Wet-spinning	-	[89]
CA	Wet-spinning	Drug delivery systems	[90]
PCL	Electrospinning	Tendon graft	[91]
GN	Electrospinning	Wound healing	[92]
CS/SF	Electrospinning	Wound healing	[93]

Abbreviations—CNW: nanocrystalline cellulose; PHBV: poly[(3-hydroxybu-tyrate)-co-(3-hydroxyvalerate)]; CS: chitosan; GN: gelatin; SA: sodium alginate; CA: cellulose acetate; SF: silk fibroin.
